# A New Assessment of Two Transferase-Based Liver Enzymes in Low- and High-Fibrosis Patients Chronically Infected with Hepatitis B Virus: A Meta-Analysis and Pilot Study

**DOI:** 10.3390/jcm13133903

**Published:** 2024-07-03

**Authors:** Marina Manea, Ion Mărunțelu, Ileana Constantinescu

**Affiliations:** 1 Immunology and Transplant Immunology, University of Medicine and Pharmacy “Carol Davila”, 020021 Bucharest, Romania; ion.maruntelu@drd.umfcd.ro (I.M.);; 2 Centre of Immunogenetics and Virology, Fundeni Clinical Institute, 022328 Bucharest, Romania

**Keywords:** HBV, chronic hepatitis, GGT, ALT, fibrosis

## Abstract

**Background:** The detection of fibrosis remains a necessity for the evaluation of hepatitis B virus (HBV)-infected patients, but the most accurate technique is invasive. Current studies aim to develop a novel noninvasive biomarker for fibrosis assessment, but no-one has found the ideal candidate. This study is a meta-analysis combined with a pilot study to investigate the connection between two transferase compounds and the levels of fibrosis. **Methods:** We studied data from PUBMED, Web of Science, and Scopus, retrieving 28,896 articles. Following PRISMA guidelines, we finally analyzed full-text articles written in English. The excluded items were duplicates, non-article entries, and irrelevant papers. We assessed the variations in alanine aminotransferase (ALT) and gamma-glutamyl transferase (GGT) levels between patients with high and low levels of fibrosis. Joanna Briggs Institute tools were used to assess article quality. We used R 4.2.2 for statistics. The pilot study included 14 randomly chosen patients with different fibrosis levels. **Results:** We found significant differences in ALT and GGT levels between patients with high and low fibrosis. The GGT/ALT ratio correlated with the levels of fibrosis and the fibrosis-4 (FIB-4) score. **Conclusions:** This meta-analysis assessed ALT and GGT levels in chronic HBV patients with fibrosis. The pilot study identified the first association between fibrosis and the GGT/ALT ratio in a Romanian cohort of chronic patients. This brings new ideas for future research.

## 1. Introduction

According to recent statistics from the World Health Organization (WHO), chronic infection with hepatitis B virus (HBV) has caused more deaths in recent years, despite global efforts to improve prevention and diagnosis [1]. A new WHO report also underlines the necessity of focusing on the more rapid and easy detection of HBV infection to reduce potential harmful and lethal complications, such as cirrhosis or hepatocellular carcinoma (HCC) [2]. 

Researchers have shown that fibrosis occurs during the natural course of chronic HBV as a response to all the liver-damaging events caused by this disease [3]. According to several authors, current guidelines recommend the assessment of liver fibrosis during the progression of HBV [2,3,4], but there is a debate regarding the most appropriate method for this. An English study evaluated multiple ways of detecting fibrosis in chronic hepatitis B (CHB) patients, and the results demonstrated the cost-effectiveness of noninvasive techniques in HBeAg-negative individuals [5]. Many authors have studied assays belonging to multiple categories [3,4,5]. According to Lai et al., fibrosis may be detected by serum biomarkers, several estimation indices, and elastography techniques [3]. The WHO recommends the use of the aspartate aminotransferase-to-platelet ratio index (APRI) and fibrosis-4 (FIB-4) scores, together with transient elastography measurements [2]. However, there is a debate related to the best technique for the noninvasive assessment of fibrosis because most known methods have limitations [3,4]. Cut-off values represent their main drawback [4]. Some methods have accuracy issues. Others are unable to distinguish between different stages of fibrosis [3].

Therefore, new prognostic noninvasive biomarkers are being studied. Some researchers believe that liver stiffness (LSM) and HBeAg detection are ideal for evaluating fibrosis in a group of patients with certain amounts of alanine aminotransferase (ALT) [6]. Others combine aspartate transferase (AST) values with gamma-glutamyl transferase (GGT) levels and viral hepatitis B core antibody (anti-HBc) levels to obtain the desired noninvasive score [7]. To find the ideal fibrosis biomarker, scientists took into consideration even more complex combinations of biochemical, hematological, viral, and patient traits. Thus, researchers envisioned scores like the one combining platelet levels, age, phosphatase, alpha-fetoprotein (AFP), and AST or, in short, PAPAS [8]. However, their performance remains modest, and the aminotransferase values limit their cut-offs. Other studies present efforts to find new predictive scores with better detection accuracy than the APRI and FIB-4 [9,10]. These either contained the values of classical biomarkers, such as platelet counts, GGT, and albumin [9], or comprised new elements such as laminin and a procollagen-based peptide [10]. However, studies are continuing on newly developed index tests, and scientists are debating their utility [3].

Overall, researchers believe that the detection of liver fibrosis leads not to one, but to a combination of noninvasive biomarkers in all chronic liver diseases [11]. Finding a suitable index score remains a challenge, with new possible candidates emerging from hematological parameters such as hemoglobin levels or the mean platelet volume [12]. Nevertheless, the most investigated biomarkers are based on GGT [3,4,13,14] or aminotransferase levels [3,4,15]. However, the current combinations of index scores fail to detect some of the fibrotic changes in chronic hepatitis B (CHB) patients [3,4,16]. Scientists emphasize that elastography also shows limitations in some populations with high viremia levels [17]. 

Another debated issue relates to the values of liver enzymes in fibrotic HBV patients. Scientists underline that ALT and GGT rise in chronic viral infections [18]. However, some researchers believe that fibrosis in HBV can occur in normal-ALT individuals [19]. A recent study also showed that HBV patients with high GGT levels and metabolic disorders are more at risk for developing fibrosis than others. However, this research was cross-sectional, and it did not track GGT variation over time [20]. 

The current study aimed to assess two biochemical markers (ALT and GGT) in high-and low-fibrosis patients with chronic HBV. We first conducted a meta-analysis to explore the differences between two of the most researched biomarkers (ALT and GGT) in patients with different levels of fibrosis. Then, we developed a small pilot study to explore our findings in Romanian patients. For the first time, to our knowledge, this pilot study explored the GGT/ALT ratio in fibrotic individuals with HBV. This work brings novelty in two main ways. First, the meta-analysis established the variations in ALT and GGT in different HBV patients with fibrosis. This effort was intended to update current knowledge related to liver enzyme values. Secondly, the pilot study explored for the first time the GGT/ALT ratio in HBV Romanian individuals with fibrosis. Our findings open new possibilities for using the GGT/ALT ratio as a noninvasive fibrosis biomarker in HBV patients.

## 2. Materials and Methods

### 2.1. The Search Process

The design of the meta-analysis was based on the Preferred Reporting Items for Systematic Review and Meta-analysis (PRISMA) guidelines [21]. Several articles were retrieved from three databases (PUBMED, Web of Science, and Scopus) using key elements such as “chronic hepatitis” (together with its derivatives and abbreviations), “patient”, “virus”, “liver”, “infection”, “fibrosis” (or “liver fibrosis”), “ALT” (and its derivatives), and “GGT” (and its derivatives). We used the Systematic Review Accelerator (https://sr-accelerator.com/, Bond University, Gold Coast, QLD, Australia; accessed on 19 June 2024) to develop the search strategy [22]. Therefore, we conducted a large-scale study analysis with 28,896 studies. These were searched for in databases from inception until 19 June 2024.

### 2.2. Article Selection

We eliminated duplicates using Systematic Review Accelerator (https://sr-accelerator.com/, Bond University, Gold Coast, QLD, Australia; accessed on 19 June 2024) [22]. A pre-written form included the entire selection process. We tracked the ALT and GGT levels in chronic HBV patients. Comparisons included individuals with low and high fibrosis scores. We eliminated records without these values, together with non-article entries such as reviews, guidelines, editorials, conference papers, letters, commentaries, and pre-prints. The exclusion criteria also included retracted articles together with non-English papers. Every included article had an accessible full text. Two independent authors (MM and IM) performed the selection process with Systematic Review Accelerator (https://sr-accelerator.com/, Bond University, Gold Coast, QLD, Australia; accessed on 19 June 2024) [22]. Discussion led to a consensus.

### 2.3. The Data Extraction Process and Quality Check

Two authors conducted an independent data extraction process (MM and IM), while the third (IC) resolved disagreements. A form included the gathered data. The main elements of interest were the study’s characteristics (author details, research type), the number and the age of the patients involved, and the ALT and GGT values. We used ZOTERO (http://www.zotero.org; accessed on 14 February 2023) [23] as an archive for the retrieved papers. The Joanna Briggs Institute (JBI; https://jbi.global/critical-appraisal-tools; accessed on 19 June 2024) [24,25] provided the critical tools for the quality check and bias assessment. Accepted articles had “Yes” answers on more than half of the items on the quality tool form.

### 2.4. Patient Selection for the Pilot Study

The pilot study included 14 random HBV patients with chronic infection from among those monitored at the Fundeni Clinical Institute, Bucharest, Romania, between 2020 and 2023. We used information regarding their biochemical and hematological status together with viremia levels. The assays were conducted in the same week as the fibrosis severity evaluation. An independent clinician performed the fibroscan measurements. These are obtained via a noninvasive technique, as described in previous studies [26]. The METAVIR score provided an interpretation of the results [27]. We included males and females over 18 with a more-than-6-month-old documented HBV infection. Pregnancy, other causes of hepatitis, and co-existing infections were not found in the included patients. The patients consented to participate in a written manner. The Ethical Council of Fundeni Clinical Institute approved our research, and we also followed the Declaration of Helsinki. 

### 2.5. Statistics

We displayed statistics using R 4.2.2 (R Foundation for Statistical Computing, Vienna, Austria) [28]. A random-effect analysis and inverse-variance weighting were used to pool standardized mean differences (SMD) for the retrieved articles. The *I*^2^ statistic, provided by Higgins and Thompson [29], helped with the heterogeneity assessment. Over 75% was considered to indicate a high heterogeneity level. Data display modalities included forest and funnel plots. Egger’s test assessed publication bias. Subgroup analysis evaluated some potential causes of heterogeneity. For the pilot study, we included medians (with interquartile ranges), means (with standard deviations), or percentages. The test used for comparisons was the Mann–Whitney test. The METAVIR scale score indicated values reflecting significant fibrosis (over F2). The Pearson test (adjusted with an FDR method) showed possible associations. Every *p*-value below 0.05 obtained in the meta-analysis and pilot study was considered significant.

## 3. Results

### 3.1. Article Selection Diagram

**Figure 1:**
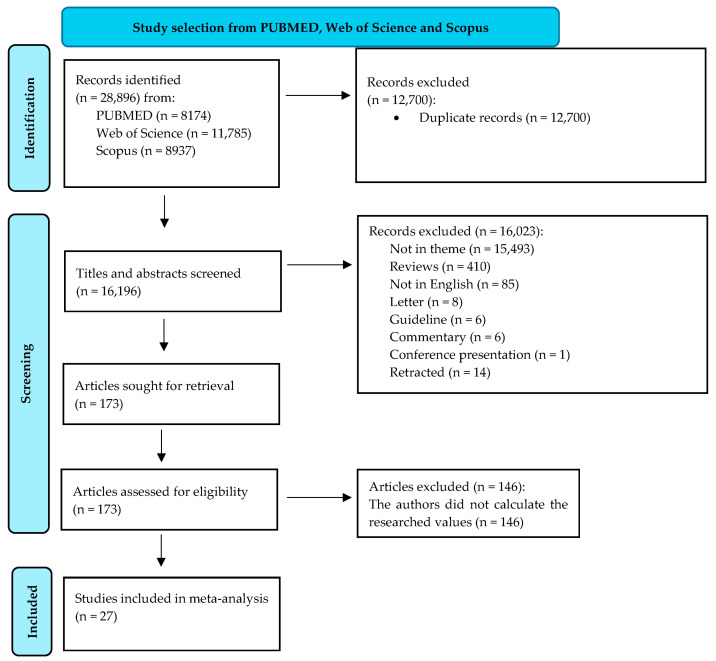
Diagram of study selection (adapted from the 2020 PRISMA Statement [21]).

The initial word search helped in the retrieval of 28,896 records. We excluded 12,700 records because they were duplicates. In total, 16,196 titles and abstracts were then screened. A thorough selection process led to the exclusion of 16,023 records. The full reasons for their elimination are presented in Figure 1. The final eligibility criteria included the presence of information related to the ALT and GGT values in chronically HBV-infected individuals and their categorization into a minimum of two categories of fibrosis carriers (low- and significant-fibrosis patients). The final meta-analysis assessed 27 articles. Figure 1 illustrates every detail of the selection process.

### 3.2. The Characteristics of the Selected Articles

Table 1 presents details regarding the included studies. 

In total, 26 articles were based on diagnostic research [30,31,32,33,34,35,36,37,38,39,41,42,43,44,45,46,47,48,49,50,51,52,53,54,55,56], and 1 was cross-sectional [40]. Most of them contained information about Chinese patients [30,31,33,34,36,37,38,39,41,42,44,46,47,49,50,51,53,54,55,56]. The authors of 17 articles [30,31,32,33,35,38,39,40,41,42,46,47,48,50,52,53,56] used the METAVIR score for the assessment of fibrosis. In seven other studies [34,36,44,49,51,53,55], researchers classified patients based on their fibrosis level using the Scheuer score. Three articles [37,43,45] contained a fibrosis assessment performed after obtaining the Ishak score. In relation to study quality, we observed that all of the included articles were well thought out. In the assessment of some articles, we even found almost perfect scores [31,33,35,36,37,39,42,53,54]. This led to the conclusion that the overall quality was good to very good, with a moderate-to-low risk of bias.

### 3.3. Results from the Meta-Analysis

**Figure 2:**
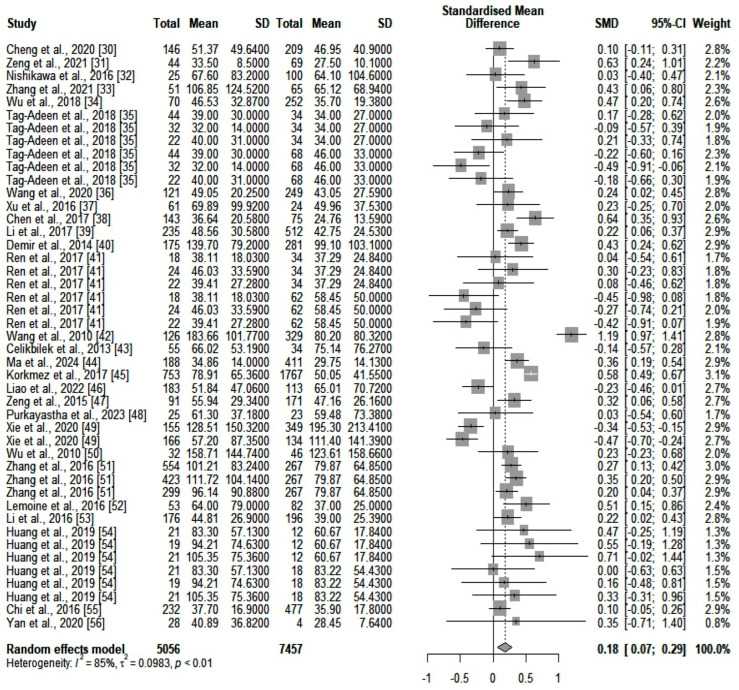
The differences in ALT values between highly fibrotic versus low-fibrosis patients.

**Figure 3:**
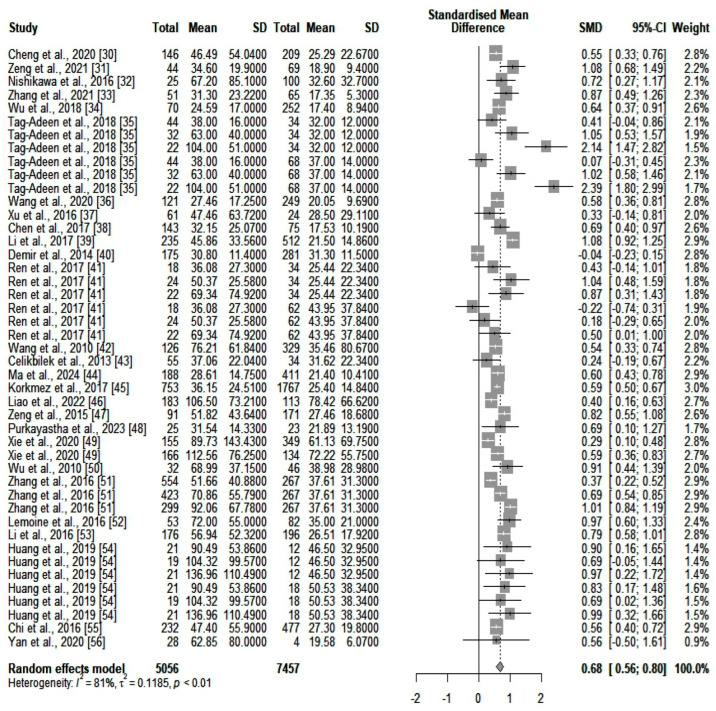
The differences in GGT values between highly fibrotic versus low-fibrosis patients. In the retrieved studies, we did not find any analyses based solely on the combination of ALT and GGT for fibrosis detection.

We compared the serum ALT values (IU/L) between the two main categories of patients (high- and low-fibrosis carriers). We identified an SMD of 0.18 (*p* = 0.0012) in favor of those who were highly fibrotic. However, heterogeneity was significant (*I*^2^ = 85%). A forest plot of our results is depicted in Figure 2. A funnel plot was also constructed (shown in Supplementary Figure S1), and an Egger’s test showed significant asymmetry in the funnel plot (*p* = 0.043). This might be consistent with publication bias. We further assessed heterogeneity with a subgroup analysis based on the number of participants included in the studies and the quality of the individual articles. Neither of the two subgroups could account for the high heterogeneity. Next, we assessed GGT (IU/L) differences between the same two categories of patients (highly fibrotic versus low-fibrosis). The results are depicted in Figure 3. The SMD value also showed a significant difference in favor of highly fibrotic patients (*p* < 0.0001). This was also accompanied by a high amount of heterogeneity (*I*^2^ = 81%). Even though the funnel plot (depicted in Supplementary Figure S2) showed a certain degree of asymmetry, the Egger’s test revealed no significant publication bias (*p* = 0.19). Subgroup analysis performed according to the number of participants and the quality of individual articles could not explain the heterogeneity.

### 3.4. The Pilot Study Results

Of our cohort of patients, 35.7% were female and 64.3% were male. The average age was 48.8 ± 14.2, with no significant difference between the high- and low-fibrosis categories. GGT was significantly higher in patients with significant fibrosis (*p* = 0.004). A considerable difference was also noticed in the platelet counts, which were considerably lower in patients with significant fibrosis. The most important difference was that in the value of the GGT/ALT index, which was almost four times bigger in individuals with high fibrosis levels by comparison with low-fibrosis persons. We also compared the new GGT/ALT index with the FIB-4 index. The latter was calculated with an online open-access algorithm [57]. A significant correlation was observed between the fibrosis level and the GGT/ALT index (r = 0.75, *p* < 0.05). A connection was also identified between the FIB-4 values and the GGT/ALT ones (r = 0.79, *p* < 0.05) (Table 2).

## 4. Discussion

Assessing fibrosis in chronic HBV infection is a challenge because, despite many possibilities, no biomarker is perfect [3]. However, it remains an important medical evaluation because it may improve decision making in the diagnosis of cirrhosis and its complications [2,58]. The gold standard assay for the progression of fibrosis in chronic liver diseases has the disadvantage of being an invasive procedure [59]. Some authors even think that it is unnecessary for HBeAg-negative patients without signs of hepatic dysfunction [60]. However, researchers believe that assessing fibrosis remains important for every chronic HBV individual [61]. Noninvasive techniques can investigate fibrosis, but their sensitivity and specificity are low [62]. Furthermore, they are affected by multiple confounders [3].

In this paper, we tried to investigate the use of two of the most studied biomarkers in fibrosis: ALT and GGT. For this reason, we conducted a meta-analysis to assess their variations in fibrotic and non-fibrotic individuals. Our first impression was that most of the retrieved studies included Asians [30,31,32,33,34,36,37,38,39,41,42,44,46,47,49,50,51,53,54,55,56], leaving the rest of the world population as potentially under-investigated. This could be an effect of the disease burden of viral hepatitis in Asia, where the WHO estimated that most infectious deaths relate to HBV [63]. Studying the consequences of HBV is important in every part of the globe for a better understanding of the viral behavior of the human host. This is mainly due to the genotypic variations of the virus in several parts of the world. Studies show that many viral genotypes might influence clinical outcomes [64,65,66]. The evolution of chronic HBV infection is also affected by other factors such as alcohol intake [67]. Researchers proved connections between environmental factors (migrations, habitat, and human activities) and the evolution of HBV genotypes and clinical outcomes [68]. A specific population genotype may influence the treatment response, mainly for interferon therapy [64]. Like other results from Far Eastern regions [69], our meta-analysis comprised articles with a predominantly young population. This study also showed significant differences in both biomarkers. Higher values were found in those with significant fibrosis. However, there was a large amount of heterogeneity, which could not be explained by the quality of the articles or by the number of persons included. Transaminase values could be affected by different treatment measures. Recent studies have shown that HBV-specific treatment influences the levels of ALT and the degree of fibrosis [70]. Another possible source of heterogeneity could be publication bias, especially for the ALT values. One also cannot neglect the fact that most of the retrieved studies did not specify the category of HBV patients taken into consideration (according to guideline classifications [71]). This could have been important because studies usually link levels of liver enzymes with the degree of liver damage [72]. Another aspect is that most of the included studies did not assess other patient comorbidities. These could have influenced liver enzyme levels, as some authors have proven [18]. 

Interestingly, the most prominent differences were observed in the GGT values from the two investigated categories of patients (SMD = 0.68 in comparison with SMD = 0.18, extracted from the ALT values). This could mean that GGT might be a candidate biomarker for fibrosis prediction. However, some authors claim that numerous non-liver-related diseases could affect the values of GGT [73]. The presence of such illnesses, which may possibly be undetected, might have caused a great amount of the heterogeneity in our study. Despite these variations, some researchers are confident in the predictive potential of GGT for illnesses such as fatty liver [74]. Research has also shown that ALT and GGT have low predictive capabilities for fibrosis if taken alone [37,43]. Because of the relatively small differences between the highly fibrotic and non-fibrotic individuals in our meta-analysis, we concluded that a combination of these two biochemical assays might be better for prediction. In our meta-analysis, we did not find any supporting evidence for an index combining ALT and GGT for fibrosis assessment. However, other scientists have investigated the GGT/ALT ratio as a potential prognostic biomarker in HBV-related hepatocellular carcinoma [75].

Therefore, we conducted a small pilot study to assess the associations among GGT, ALT, and fibrosis in a Romanian patient cohort. This was important, especially because we did not find any representative European cohort in our meta-analysis. Our findings informed the conclusions drawn from the meta-analysis. The difference in the GGT levels was larger than that in the ALT values between the two categories of patients. The difference was even bigger for the GGT/ALT combination index. This was also highly correlated with the degree of fibrosis and the FIB-4 values, showing the potential of the GGT/ALT ratio for fibrosis predictions. 

Studies prove that other biomarker combinations, such as the APRI, have low sensitivity and specificity in the assessment of fibrosis [35,45,76]. Some authors believe that the GPR (gamma-glutamyl transferase-to-platelet ratio index) could be more accurate than the APRI in the diagnosis of fibrosis, but this issue is debatable [77]. Others show that the performance of noninvasive biomarkers may vary depending on the phase of fibrosis and the patient’s country of origin [52]. Because of the small patient sample, we could not investigate the exact accuracy of the GGT/ALT ratio for fibrosis predictions. Therefore, further studies with larger patient samples should analyze this new ratio. For this purpose, several countries and centers should participate, and other noninvasive and gold-standard techniques for fibrosis evaluation should be compared with the GGT/ALT ratio. 

However, our study has several limitations. Firstly, our meta-analysis could have contained treated HBV patients, which might have influenced the obtained results. Secondly, we could not find studies aiming to investigate the possible correlation between the GGT/ALT ratio and fibrosis. This could have been useful for a broader understanding. Further multicenter studies are needed to investigate the accuracy of the GGT/ALT ratio for the evaluation of fibrosis by comparison with other known biomarkers. Another limitation is related to the size of our pilot study, which could have biased our results. However, the meta-analysis showed that the patient counts did not influence the comparisons of GGT and ALT levels between fibrotic and non-fibrotic individuals to a great extent. 

This study is important because it presents the values of two liver enzymes according to various levels of fibrosis. It also underlines for the first time the possibility of a novel noninvasive fibrosis index based only on ALT and GGT. This could have future benefits for finding the best serum assay to evaluate the progression of HBV in chronically infected patients.

## 5. Conclusions

The results presented in this study are important considering the limitations of the noninvasive biomarkers for fibrosis. Future studies could assess the accuracy of the GGT/ALT ratio by comparison with other techniques. Romanian HBV individuals could be investigated to compare the values of their liver enzymes with those of other populations. This could explain the differences in outcomes related to genotype variations in chronic HBV.

## Figures and Tables

**Table 1:** Study details.

First Author, Publication Year; Reference	Country	Study Type	Low Fibrosis Category *				High Fibrosis Category *				JBI Score *
			Participant Count	Participant Age (Years) *	ALT (IU/L)	GGT (IU/L)	Participant Count	Participant Age (Years) *	ALT (IU/L)	**GGT (IU/L)**	
Cheng et al., 2020 [30]	China	Diagnostic	209	38.8 ± 10.54	46.95 ± 40.90	25.29 ± 22.67	146	41.16 ± 9.18	51.37 ± 49.64	46.49 ± 54.04	8/10
Zeng et al., 2021 [31]	China	Diagnostic	69	36.6 ± 9.1	27.5 ± 10.1	18.9 ± 9.4	44	39.5 ± 9.4	33.5 ± 8.5	34.6 ± 19.9	9/10
Nishikawa et al., 2016 [32]	Japan	Diagnostic	100	45.5 ± 13.1	64.1 ± 104.6	32.6 ± 32.7	25	47.5 ± 11.9	67.6 ± 83.2	67.2 ± 85.1	8/10
Zhang et al., 2021 [33]	China	Diagnostic	65	-	65.12 ± 68.94	17.35 ± 5.3	51	-	106.85 ± 124.52	31.3 ± 23.22	9/10
Wu et al., 2018 [34]	China	Diagnostic	252	34.3 ± 10.44	35.7 ± 19.38	17.4 ± 8.94	70	37.52 ± 10.43	46.53 ± 32.87	24.59 ± 17	7/10
Tag-Adeen et al., 2018 [35]	Egypt	Diagnostic	Group 1: 34 Group 2: 68	Group 1: 29.5 ± 5 Group 2: 36 ± 6.7	Group 1: 34 ± 27 Group 2: 46 ± 33	Group 1: 32 ± 12 Group 2: 37 ± 14	Group 3: 44 Group 4: 32 Group 5: 22	Group 3: 36 ± 6.7 Group 4: 36.3 ± 9.8 Group 5: 39.1 ± 7	Group 3: 39 ± 30 Group 4: 32 ± 14 Group 5: 40 ± 31	Group 3: 38 ± 16 Group 4: 63 ± 40 Group 5: 104 ± 51	9/10
Wang et al., 2020 [36]	China	Diagnostic	249	33.7 ± 10.44	43.05 ± 27.59	20.05 ± 9.69	121	37.18 ± 10.87	49.05 ± 20.25	27.46 ± 17.25	9/10
Xu et al., 2016 [37]	China	Diagnostic	24	36.33 ± 7.86	49.96 ± 37.53	28.5 ± 29.11	61	38.82 ± 8.33	69.89 ± 99.92	47.46 ± 63.72	9/10
Chen et al., 2017 [38]	China	Diagnostic	75	34 ± 7.55	24.76 ± 13.59	17.53 ± 10.19	143	40 ± 8.98	36.64 ± 20.58	32.15 ± 25.07	8/10
Li et al., 2017 [39]	China	Diagnostic	512	34.7 ± 10.4	42.75 ± 24.53	21.5 ± 14.86	235	39.7 ± 13.42	48.56 ± 30.58	45.86 ± 33.56	9/10
Demir et al., 2014 [40]	Turkey	Cross-sectional	281	34.1 ± 11.5	99.1 ± 103.1	31.3 ± 11.5	175	40.9 ± 12	139.7 ± 79.2	30.8 ± 11.4	6/8
Ren et al., 2017 [41]	China	Diagnostic	Group 1: 34 Group 2: 62	Group 1: 35.21 ± 10.02 Group 2: 39.56 ± 12.12	Group 1: 37.29 ± 24.84 Group 2: 58.45 ± 50	Group 1: 25.44 ± 22.34 Group 2: 43.95 ± 37.84	Group 3: 18 Group 4: 24 Group 5: 22	Group 3: 44.68 ± 9.92 Group 4: 39.63 ± 12.36 Group 5: 39.22 ± 12.56	Group 3: 38.11 ± 18.03 Group 4: 46.03 ± 33.59 Group 5: 39.41 ± 27.28	Group 3: 36.08 ± 27.3 Group 4: 50.37 ± 25.58 Group 5: 69.34 ± 74.92	8/10
Wang et al., 2010 [42]	China	Diagnostic	329	29.44 ± 7.6	80.2 ± 80.32	35.46 ± 80.67	126	40.58 ± 11.24	183.66 ± 101.77	76.21 ± 61.84	9/10
Celikbelik et al., 2013 [43]	Turkey	Diagnostic	34	40.2 ± 11.7	75.14 ± 76.27	31.62 ± 22.34	55	42.2 ± 13.7	66.02 ± 53.19	37.06 ± 22.04	8/10
Ma et al., 2024 [44]	China	Diagnostic	411	36.7 ± 8.92	29.75 ± 14.13	21.4 ± 10.41	188	37.35 ± 8.21	34.86 ± 14	28.61 ± 14.75	6/10
Korkmez et al., 2017 [45]	Turkey	Diagnostic	1767	39.29 ± 11.92	50.05 ± 41.55	25.4 ± 14.84	753	43.67 ± 12.76	78.91 ± 65.36	36.15 ± 24.51	6/10
Liao et al., 2022 [46]	China	Diagnostic	113	45.15 ± 14.97	65.01 ± 70.72	78.42 ± 66.62	183	43.63 ± 13.32	51.84 ± 47.06	106.5 ± 73.21	6/10
Zeng et al., 2015 [47]	China	Diagnostic	171	35.8 ± 11	47.16 ± 26.16	27.46 ± 18.68	91	35.8 ± 10.6	55.94 ± 29.34	51.82 ± 43.64	7/10
Purkayastha et al., 2023 [48]	India	Diagnostic	23	28.96 ± 11.91	59.48 ± 73.38	21.89 ± 13.19	25	37.52 ± 17.82	61.3 ± 37.18	31.54 ± 14.33	6/10
Xie et al., 2020 [49]	China	Diagnostic	Group 1: 349 Group 2: 134	Group 1: 33.23 ± 9.95 Group 2: 41.55 ± 12.83	Group 1: 195.3 ± 213.41 Group 2: 111.4 ± 141.39	Group 1: 61.13 ± 69.75 Group 2: 72.22 ± 55.75	Group 1: 155 Group 2: 166	Group 1: 44.88 ± 12.22 Group 2: 53.14 ± 11.26	Group 1: 128.51 ± 150.32 Group 2: 57.2 ± 87.35	Group 1: 89.73 ± 143.43 Group 2: 112.56 ± 76.25	7/10
Wu et al., 2010 [50]	China	Diagnostic	46	29.6 ± 12	123.61 ± 158.66	38.98 ± 28.98	32	36.9 ± 11.4	158.71 ± 144.74	68.99 ± 37.15	7/10
Zhang et al., 2016 [51]	China	Diagnostic	267	29.43 ± 9.09	79.87 ± 64.85	37.61 ± 31.3	Group 3: 554 Group 4: 423 Group 5: 299	Group 3: 30.55 ± 9.23 Group 4: 31.22 ± 9.11 Group 5: 35.79 ± 10.8	Group 3: 101.21 ± 83.24 Group 4: 111.72 ± 104.14 Group 5: 96.14 ± 90.88	Group 3: 51.66 ± 40.88 Group 4: 70.86 ± 55.79 Group 5: 92.06 ± 67.78	10/10
Lemoine et al., 2016 [52]	Gambia	Diagnostic	82	36 ± 10	37 ± 25	35 ± 21	53	35 ± 11	64 ± 79	72 ± 55	7/10
Li et al., 2016 [53]	China	Diagnostic	196	38 ± 11	39 ± 25.39	26.51 ± 17.92	176	40 ± 12	44.81 ± 26.9	56.94 ± 52.32	9/10
Huang et al., 2019 [54]	China	Diagnostic	Group 1: 12 Group 2: 18	Group 1: 32.25 ± 10.36 Group 2: 38.17 ± 10.99	Group 1: 60.67 ± 17.84 Group 2: 83.22 ± 54.43	Group 1: 46.5 ± 32.95 Group 2: 55.53 ± 38.34	Group 3: 21 Group 4: 19 Group 5: 21	Group 3: 40.9 ± 11.45 Group 4: 43.68 ± 12.37 Group 5: 46.33 ± 13.46	Group 3: 83.3 ± 57.13 Group 4: 94.21 ± 74.63 Group 5: 105.35 ± 75.36	Group 3: 90.49 ± 53.86 Group 4: 104.32 ± 99.57 Group 5: 136.96 ± 110.49	9/10
Chi et al., 2016 [55]	China	Diagnostic	477	37.9 ± 9.7	35.9 ± 17.8	27.3 ± 19.8	432	42.2 ± 10.6	37.7 ± 16.9	47.4 ± 55.9	6/10
Yan et al., 2020 [56]	China	Diagnostic	4	45.5 ± 5.97	28.45 ± 7.64	19.58 ± 6.07	28	46.18 ± 10.4	40.89 ± 36.82	62.85 ± 80	7/10

* Data are presented as mean ± standard deviation (SD). The JBI score is presented as the ratio between the number of “Yes” answers to the questions on the critical appraisal tool and the total number of possible “Yes” answers. ALT—alanine aminotransferase, GGT—gamma glutamyl transferase, JBI—Joanna Briggs Institute.

**Table 2:** The differences between the two main categories of patients.

Variable	Total (n = 14)	Low or no Fibrosis (n = 10)	Significant Fibrosis (n = 4)	*p*-Value **
Age (years)	48.8 ± 14.2	46.8 ± 15.4	53.8 ± 10.9	0.41
Sex-female (%)	5 (35.7)	3 (30)	2 (50)	0.92
ALT * (IU/L)	41 ± 25.6	45.9 ± 27.3	28.8 ± 18	0.25
AST * (IU/L)	28.6 ± 10.1	27.2 ± 9.1	32 ± 13	0.43
GGT * (IU/L)	41.1 ± 37	26.7 ± 10.4	77 ± 56.7	0.004
GGT/ALT index	1.2 ± 1.2	0.6 ± 0.2	2.6 ± 1.6	<0.0001
Leucocyte count (×10^3^ µL)	7.2 ± 3	7.8 ± 3.3	5.7 ± 1.5	0.22
Platelet count (×10^3^ µL)	219.6 ± 58.6	239.2 ± 53.1	170.8 ± 44.4	0.02
HBV-DNA (IU/mL)	394.5 [81.5, 4687.2]	548.5 [81.5, 4687.2]	394.5 [250.5, 13,697.5]	0.88

* ALT—alanine aminotransferase, AST—aspartate aminotransferase, GGT—gamma glutamyl transferase; ** *p*-value < 0.05 was considered significant.

## Data Availability

The original contributions presented in the study are included in the article/Supplementary Materials, further inquiries can be directed to the corresponding author.

## Supplementary Materials

The following supporting information can be downloaded at https://www.mdpi.com/article/10.3390/jcm13133903/s1: Figure S1: Funnel plot of ALT values (IU/L), Figure S2: Funnel plot of GGT values (IU/L).

## References

[1] WHO Sounds Alarm on Viral Hepatitis Infections Claiming 3500 Lives Each Day. https://www.who.int/news/item/09-04-2024-who-sounds-alarm-on-viral-hepatitis-infections-claiming-3500-lives-each-day.

[2] Consolidated Guidelines on Person-Centred Viral Hepatitis Strategic Information. https://www.who.int/publications/i/item/9789240091313.

[3] Lai J.C.-T., Liang L.Y., Wong G.L.-H. (2024). Noninvasive tests for liver fibrosis in 2024: Are there different scales for different diseases?. Gastroenterol. Rep..

[4] Bera C., Hamdan-Perez N., Patel K. (2024). Non-Invasive Assessment of Liver Fibrosis in Hepatitis B Patients. J. Clin. Med..

[5] Crossan C., Tsochatzis E.A., Longworth L., Gurusamy K., Papastergiou V., Thalassinos E., Mantzoukis K., Rodriguez-Peralvarez M., O’Brien J., Noel-Storr A. (2016). Cost-effectiveness of noninvasive liver fibrosis tests for treatment decisions in patients with chronic hepatitis B in the UK: Systematic review and economic evaluation. J. Viral Hepat..

[6] Chen Y., Li Y., Li N., Fan X., Li C., Zhang P., Han Q., Liu Z. (2018). A noninvasive score to predict liver fibrosis in HBeAg-positive hepatitis B patients with normal or minimally elevated alanine aminotransferase levels. Dis. Markers.

[7] Chen S., Huang H., Huang W. (2021). A noninvasive model to predict liver histology for antiviral therapy decision in chronic hepatitis B with alanine aminotransferase < 2 upper limit of normal. BMC Gastroenterol..

[8] Seto W.-K., Lee C.-F., Lai C.-L., Ip P.P.C., Fong Y.-T., Fung J., Wong K.-H., Yuen M.-F. (2011). A new model using routinely available clinical parameters to predict significant liver fibrosis in chronic hepatitis B. PLoS ONE.

[9] Kang N.-L., Ruan Q.-F., Zhang D.-S., Yu X.-P., Hu Z.-T., Lin Z.-M., Wu L.-Y., Lin M.-X., Huang Z.-X., Jiang J.-J. (2022). Advantages of a Novel Model for Predicting Hepatic Fibrosis in Chronic Hepatitis B Virus Carriers Compared with APRI and FIB-4 Scores. J. Clin. Transl. Hepatol..

[10] Chen Y., Gong J., Zhou W., Jie Y., Li Z., Chong Y., Hu B. (2020). A Novel Prediction Model for Significant Liver Fibrosis in Patients with Chronic Hepatitis B. BioMed Res. Int..

[11] Calès P., Oberti F., Michalak S., Hubert-Fouchard I., Rousselet M.-C., Konaté A., Gallois Y., Ternisien C., Chevallier A., Lunel F. (2005). A novel panel of blood markers to assess the degree of liver fibrosis. Hepatology.

[12] Nyarko E.N.Y., Obirikorang C., Owiredu W.K.B.A., Adu E.A., Acheampong E. (2023). Assessment of the performance of haematological and non-invasive fibrotic indices for the monitoring of chronic HBV infection: A pilot study in a Ghanaian population. BMC Res. Notes.

[13] Ding R., Lu W., Zhou X., Huang D., Wang Y., Li X., Li Y., Lin W., Song S., Zhang Z. (2021). A Novel Non-invasive Model Based on GPR for the Prediction of Liver Fibrosis in Patients With Chronic Hepatitis B. Front. Med..

[14] Wang R.-Q., Zhang Q.-S., Zhao S.-X., Niu X.-M., Du J.-H., Du H.-J., Nan Y.-M. (2016). Gamma-glutamyl transpeptidase to platelet ratio index is a good noninvasive biomarker for predicting liver fibrosis in Chinese chronic hepatitis B patients. J. Int. Med. Res..

[15] Sanai F.M., Farah T., Albeladi K., Batwa F., Dahlan Y., Babatin M.A., Al-Ashgar H., AlMana H., Alsaad K.S., AlSwat K. (2017). Diminished accuracy of biomarkers of fibrosis in low replicative chronic hepatitis B. BMC Gastroenterol..

[16] Huang R., Wang G., Tian C., Liu Y., Jia B., Wang J., Yang Y., Li Y., Sun Z., Yan X. (2017). Gamma-glutamyl-Transpeptidase to platelet ratio is not superior to APRI,FIB-4 and RPR for diagnosing liver fibrosis in CHB patients in China. Sci. Rep..

[17] Mbaye P.S., Sarr A., Sire J.-M., Evra M.-L., Ba A., Daveiga J., Diallo A., Fall F., Chartier L., Simon F. (2011). Liver stiffness measurement and biochemical markers in senegalese chronic hepatitis B patients with normal ALT and high viral load. PLoS ONE.

[18] Kalas M.A., Chavez L., Leon M., Taweesedt P.T., Surani S. (2021). Abnormal liver enzymes: A review for clinicians. World J. Hepatol..

[19] Wei S., Xie Q., Liao G., Chen H., Hu M., Lin X., Li H., Peng J. (2024). Patients with chronic hepatitis B who have persistently normal alanine aminotransferase or aged < 30 years may exhibit significant histologic damage. BMC Gastroenterol..

[20] Lv H., Jiang Y., Zhu G., Liu S., Wang D., Wang J., Zhao K., Liu J. (2023). Liver fibrosis is closely related to metabolic factors in metabolic associated fatty liver disease with hepatitis B virus infection. Sci. Rep..

[21] Page M.J., Moher D., Bossuyt P.M., Boutron I., Hoffmann T.C., Mulrow C.D., Shamseer L., Tetzlaff J.M., Akl E.A., Brennan S.E. (2021). PRISMA 2020 explanation and elaboration: Updated guidance and exemplars for reporting systematic reviews. BMJ.

[22] Clark J., Glasziou P., Del Mar C., Bannach-Brown A., Stehlik P., Scott A.M. (2020). A full systematic review was completed in 2 weeks using automation tools: A case study. J. Clin. Epidemiol..

[23] ZOTERO. http://www.zotero.org.

[24] Whiting P.F., Rutjes A.W., Westwood M.E., Mallett S., Deeks J.J., Reitsma J.B., Leeflang M.M., Sterne J.A., Bossuyt P.M., QUADAS-2 Group (2011). QUADAS-2: A revised tool for the quality assessment of diagnostic accuracy studies. Ann. Intern. Med..

[25] Campbell J.M., Klugar M., Ding S., Carmody D.P., Hakonsen S.J., Jadotte Y.T., White S., Munn Z. (2015). Diagnostic test accuracy: Methods for systematic review and meta-analysis. Int. J. Evid. Based Healthc..

[26] Patel K., Wilder J. (2014). Fibroscan. Clin. Liver Dis..

[27] Osman A.M., El Shimy A., Abd El Aziz M.M. (2020). 2D shear wave elastography (SWE) performance versus vibration-controlled transient elastography (VCTE/fibroscan) in the assessment of liver stiffness in chronic hepatitis. Insights Imaging.

[28] R Core Team (2022). R: A Language and Environment for Statistical Computing.

[29] Higgins J.P.T., Thompson S.G. (2002). Quantifying heterogeneity in a meta-analysis. Stat. Med..

[30] Cheng D., Wan G., Sun L., Wang X., Ou W., Xing H. (2020). A Novel Diagnostic Nomogram for Noninvasive Evaluating Liver Fibrosis in Patients with Chronic Hepatitis B Virus Infection. BioMed Res. Int..

[31] Zeng D.-W., Huang Z.-X., Lin M.-X., Kang N.-L., Lin X., Li Y.-N., Zhu Y.-Y., Liu Y.-R. (2021). A novel HBsAg-based model for predicting significant liver fibrosis among Chinese patients with immune-tolerant phase chronic hepatitis B: A multicenter retrospective study. Ther. Adv. Gastroenterol..

[32] Nishikawa H., Hasegawa K., Ishii A., Takata R., Enomoto H., Yoh K., Kishino K., Shimono Y., Iwata Y., Nakano C. (2016). A proposed predictive model for advanced fibrosis in patients with chronic hepatitis B and its validation. Medicine.

[33] Zhang K.-L., Chen X.-Q., Lv Z.-L., Tang Q., Shan Q.-W., Enomoto H. (2021). A simple noninvasive model to predict significant fibrosis in children with chronic hepatitis B. Medicine.

[34] Wu X., Cai B., Su Z., Li Y., Xu J., Deng R., Wang L. (2018). Aspartate transaminase to platelet ratio index and gamma-glutamyl transpeptidase-to-platelet ratio outweigh fibrosis index based on four factors and red cell distribution width-platelet ratio in diagnosing liver fibrosis and inflammation in chronic hepatitis B. J. Clin. Lab. Anal..

[35] Tag-Adeen M., Omar M.Z., Abd-Elsalam F.M., Hasaneen A., Mohamed M.A., Elfeky H.M., Said E.M., Abdul-Aziz B., Osman A.H., Ahmed E.S. (2018). Assessment of liver fibrosis in Egyptian chronic hepatitis B patients: A comparative study including 5 noninvasive indexes. Medicine.

[36] Wang L., Li J., Yang K., Zhang H., Wang Q., Lv X., Guan S. (2020). Comparison and evaluation of non-invasive models in predicting liver inflammation and fibrosis of chronic hepatitis B virus-infected patients with high hepatitis B virus DNA and normal or mildly elevated alanine transaminase levels. Medicine.

[37] Xu S.-H., Li Q., Hu Y.-P., Ying L. (2016). Development of a model based on biochemical, real-time tissue elastography and ultrasound data for the staging of liver fibrosis and cirrhosis in patients with chronic hepatitis B. Mol. Med. Rep..

[38] Chen X., Wen H., Zhang X., Dong C., Lin H., Guo Y., Shan L., Yao S., Yang M., Le X. (2017). Development of a Simple Noninvasive Model to Predict Significant Fibrosis in Patients with Chronic Hepatitis B: Combination of Ultrasound Elastography, Serum Biomarkers, and Individual Characteristics. Clin. Transl. Gastroenterol..

[39] Li Q., Lu C., Li W., Huang Y., Chen L. (2017). Evaluation of eLIFT for Non-invasive Assessment of Liver fibrosis and Cirrhosis in Patients with Chronic Hepatitis B Virus Infection. Sci. Rep..

[40] Demir N.A., Kolgelier S., Ozcimen S., Gungor G., Sumer S., Demir L.S., Inkaya A.C., Ural O. (2014). Evaluation of the relation between hepatic fibrosis and basic laboratory parameters in patients with chronic hepatitis B fibrosis and basic laboratory parameters. Hepat. Mon..

[41] Ren T., Wang H., Wu R., Niu J. (2017). Gamma-glutamyl transpeptidase-to-platelet ratio predicts significant liver fibrosis of chronic Hepatitis B patients in China. Gastroenterol. Res. Pract..

[42] Wang D., Wang Q., Shan F., Liu B., Lu C. (2010). Identification of the risk for liver fibrosis on CHB patients using an artificial neural network based on routine and serum markers. BMC Infect. Dis..

[43] Celikbilek M., Dogan S., Gursoy S., Zararsiz G., Yurci A., Ozbakir O., Guven K., Yucesoy M. (2013). Noninvasive assessment of liver damage in chronic hepatitis B. World J. Hepatol..

[44] Ma S., Zhou L., Lin S., Li M., Luo J., Chen L. (2024). Noninvasive Models to Assess Liver Inflammation and Fibrosis in Chronic HBV Infected Patients with Normal or Mildly Elevated Alanine Transaminase Levels: Which One Is Most Suitable?. Diagnostics.

[45] Korkmaz P., Demirturk N., Batırel A., Cem Yardimci A., Cagir U., Nemli S.A., Korkmaz F., Akcam F.Z., Barut H.S., Bayrak B. (2017). Noninvasive models to predict liver fibrosis in patients with chronic hepatitis B: A study from Turkey. Hepat. Mon..

[46] Liao M.-J., Li J., Dang W., Chen D.-B., Qin W.-Y., Chen P., Zhao B.-G., Ren L.-Y., Xu T.-F., Chen H.-S. (2022). Novel index for the prediction of significant liver fibrosis and cirrhosis in chronic hepatitis B patients in China. World J. Gastroenterol..

[47] Zeng X., Xu C., He D., Li M., Zhang H., Wu Q., Xiang D., Wang Y. (2015). Performance of several simple, noninvasive models for assessing significant liver fibrosis in patients with chronic hepatitis B. Croat. Med. J..

[48] Purkayastha S., Jha A., Kumar R., Dayal V., Jha S. (2023). Serum Gamma-Glutamyl Transpeptidase-to-Platelet Ratio as a Noninvasive Marker of Liver Fibrosis in Chronic Hepatitis B. Cureus J. Med. Sci..

[49] Xie G., Wang X., Wei R., Wang J., Zhao A., Chen T., Wang Y., Zhang H., Xiao Z., Liu X. (2020). Serum metabolite profiles are associated with the presence of advanced liver fibrosis in Chinese patients with chronic hepatitis B viral infection. BMC Med..

[50] Wu S.-D., Wang J.-Y., Li L. (2010). Staging of liver fibrosis in chronic hepatitis B patients with a composite predictive model: A comparative study. World J. Gastroenterol..

[51] Zhang Z., Wang G., Kang K., Wu G., Wang P. (2016). The diagnostic accuracy and clinical utility of three noninvasive models for predicting liver fibrosis in patients with HBV infection. PLoS ONE.

[52] Lemoine M., Shimakawa Y., Nayagam S., Khalil M., Suso P., Lloyd J., Goldin R., Njai H.-F., Ndow G., Taal M. (2016). The gamma-glutamyl transpeptidase to platelet ratio (GPR) predicts significant liver fibrosis and cirrhosis in patients with chronic HBV infection in West Africa. Gut.

[53] Li Q., Song J., Huang Y., Li X., Zhuo Q., Li W., Chen C., Lu C., Qi X., Chen L. (2016). The gamma-glutamyl-transpeptidase to platelet ratio does not show advantages than APRI and fib-4 in diagnosing significant fibrosis and cirrhosis in patients with chronic hepatitis B A retrospective cohort study in China. Medicine.

[54] Huang D., Lin T., Wang S., Cheng L., Xie L., Lu Y., Chen M., Zhu L., Shi J. (2019). The liver fibrosis index is superior to the APRI and FIB-4 for predicting liver fibrosis in chronic hepatitis B patients in China. BMC Infect. Dis..

[55] Chi X.-L., Shi M.-J., Xiao H.-M., Xie Y.-B., Cai G.-S. (2016). The score model containing Chinese medicine syndrome element of blood stasis presented a better performance compared to APRI and FIB-4 in diagnosing advanced fibrosis in patients with chronic hepatitis B. Evid.-Based Complement. Altern. Med..

[56] Yan L.-T., Wang L.-L., Yao J., Yang Y.-T., Mao X.-R., Yue W., Mao Y.-W., Zhou W., Chen Q.-F., Chen Y. (2020). Total bile acid-to-cholesterol ratio as a novel noninvasive marker for significant liver fibrosis and cirrhosis in patients with non-cholestatic chronic hepatitis B virus infection. Medicine.

[57] Fibrosis-4 (FIB-4) Index for Liver Fibrosis. https://www.mdcalc.com/calc/2200/fibrosis-4-fib-4-index-liver-fibrosis.

[58] Castera L. (2014). Hepatitis B: Are non-invasive markers of liver fibrosis reliable?. Liver Int..

[59] Larrey D., Meunier L., Ursic-Bedoya J. (2017). Liver Biopsy in Chronic Liver Diseases: Is There a Favorable Benefit: Risk Balance?. Ann. Hepatol..

[60] Papatheodoridis G.V., Manolakopoulos S., Liaw Y.-F., Lok A. (2012). Follow-up and indications for liver biopsy in HBeAg-negative chronic hepatitis B virus infection with persistently normal ALT: A systematic review. J. Hepatol..

[61] Chao D.T., Lim J.K., Ayoub W.S., Nguyen L.H., Nguyen M.H. (2014). Systematic review with meta-analysis: The proportion of chronic hepatitis B patients with normal alanine transaminase ≤40 IU/L and significant hepatic fibrosis. Aliment. Pharmacol. Ther..

[62] Johannessen A., Stockdale A.J., Henrion M.Y.R., Okeke E., Seydi M., Wandeler G., Sonderup M., Spearman C.W., Vinikoor M., Sinkala E. (2023). Systematic review and individual-patient-data meta-analysis of non-invasive fibrosis markers for chronic hepatitis B in Africa. Nat. Commun..

[63] Hepatitis. South-East Asia. https://www.who.int/southeastasia/health-topics/hepatitis.

[64] Fernandes da Silva C., Keeshan A., Cooper C. (2023). Hepatitis B virus genotypes influence clinical outcomes: A review. Can. Liver J..

[65] Raihan R., Akbar S.M.F. (2023). A Narrative Review on the Specific Pattern of HBV Genotype in Bangladesh: Clinical Implications for Management. Euroasian J. Hepato-Gastroenterol..

[66] Al-Sadeq D.W., Taleb S.A., Zaied R.E., Fahad S.M., Smatti M.K., Rizeq B.R., Al Thani A.A., Yassine H.M., Nasrallah G.K. (2019). Hepatitis B Virus Molecular Epidemiology, Host-Virus Interaction, Coinfection, and Laboratory Diagnosis in the MENA Region: An Update. Pathogens.

[67] Ganesan M., Eikenberry A., Poluektova L.Y., Kharbanda K.K., Osna N.A. (2020). Role of alcohol in pathogenesis of hepatitis B virus infection. World J. Gastroenterol..

[68] Roman S., Jose-Abrego A., Fierro N.A., Escobedo-Melendez G., Ojeda-Granados C., Martinez-Lopez E., Panduro A. (2014). Hepatitis B virus infection in Latin America: A genomic medicine approach. World J. Gastroenterol..

[69] Ie S.I., Thedja M.D., Sidarta E., Purnomo G.A., Turyadi, Pattiiha M.Z., Muljono D.H., Sadhewa A., Harahap A.R., Soedarmono Y.S.M. (2015). High Prevalence of Hepatitis B Virus Infection in Young Adults in Ternate, Eastern Indonesia. Am. J. Trop. Med. Hyg..

[70] Lim Y.-S., Kim W.R., Dieterich D., Kao J.-H., Flaherty J.F., Yee L.J., Roberts L.R., Razavi H., Kennedy P.T.F. (2023). Evidence for Benefits of Early Treatment Initiation for Chronic Hepatitis B. Viruses.

[71] Wong G.L.-H. (2018). Management of chronic hepatitis B patients in immunetolerant phase: What latest guidelines recommend. Clin. Mol. Hepatol..

[72] Kennedy P.T.F., Litwin S., Dolman G., Bertoletti A., Mason W. (2017). Immune Tolerant Chronic Hepatitis B: The Unrecognized Risks. Viruses.

[73] Kunutsor S.K. (2016). Gamma-glutamyltransferase—Friend or foe within?. Liver Int..

[74] Fujii H., Doi H., Ko T., Fukuma T., Kadono T., Asaeda K., Kobayashi R., Nakano T., Doi T., Nakatsugawa Y. (2020). Frequently abnormal serum gamma-glutamyl transferase activity is associated with future development of fatty liver: A retrospective cohort study. BMC Gastroenterol..

[75] Zhao Z., Zhu Y., Ni X., Lin J., Li H., Zheng L., Zhang C., Qi X., Huo H., Lou X. (2021). Serum GGT/ALT ratio predicts vascular invasion in HBV-related HCC. Cancer Cell Int..

[76] Xy X., Wang W.S., Zhang Q.M., Li J.L., Sun J.B., Qin T.T., Liu H.B. (2019). Performance of common imaging techniques vs serum biomarkers in assessing fibrosis in patients with chronic hepatitis B: A systematic review and meta-analysis. World J. Clin. Cases.

[77] Wu J., Mao W. (2020). Review of Serum Biomarkers and Models Derived from Them in HBV-Related Liver Diseases. Dis. Markers.

