# Supplementary figures and images for: A New Assessment of Two Transferase-Based Liver Enzymes in Low- and High-Fibrosis Patients Chronically Infected with Hepatitis B Virus: A Meta-Analysis and Pilot Study
Source: J Clin Med. 2024 Jul 3;13(13):3903. doi: 10.3390/jcm13133903 (PMC11242663; doi:10.3390/jcm13133903)

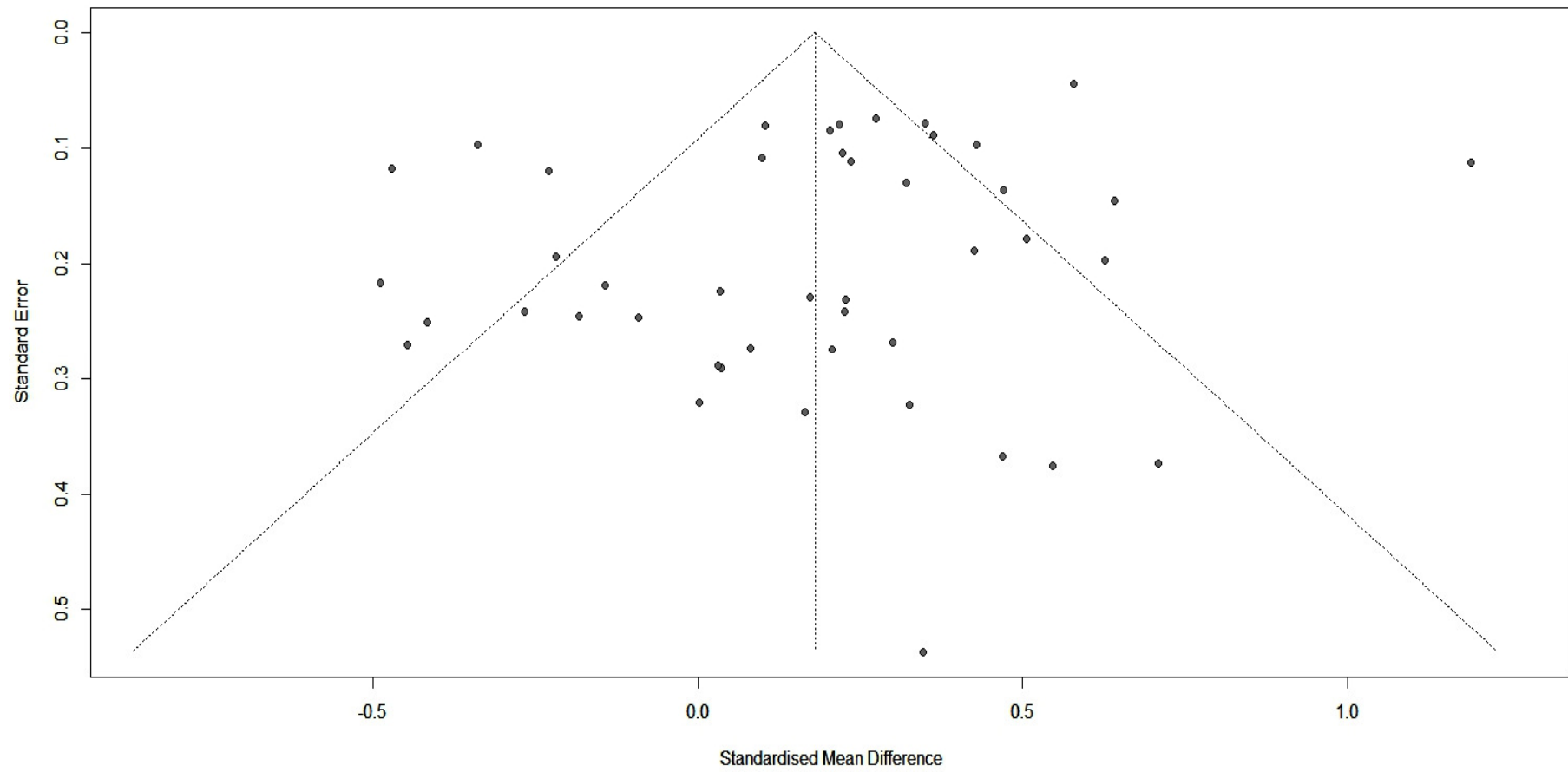

**Supplementary Figure S1:** Funnel plot of ALT values (IU/L)

Supplement: Supplementary file 1 [file jcm-13-03903-s001.zip › Supplementary Figure S1.pdf]

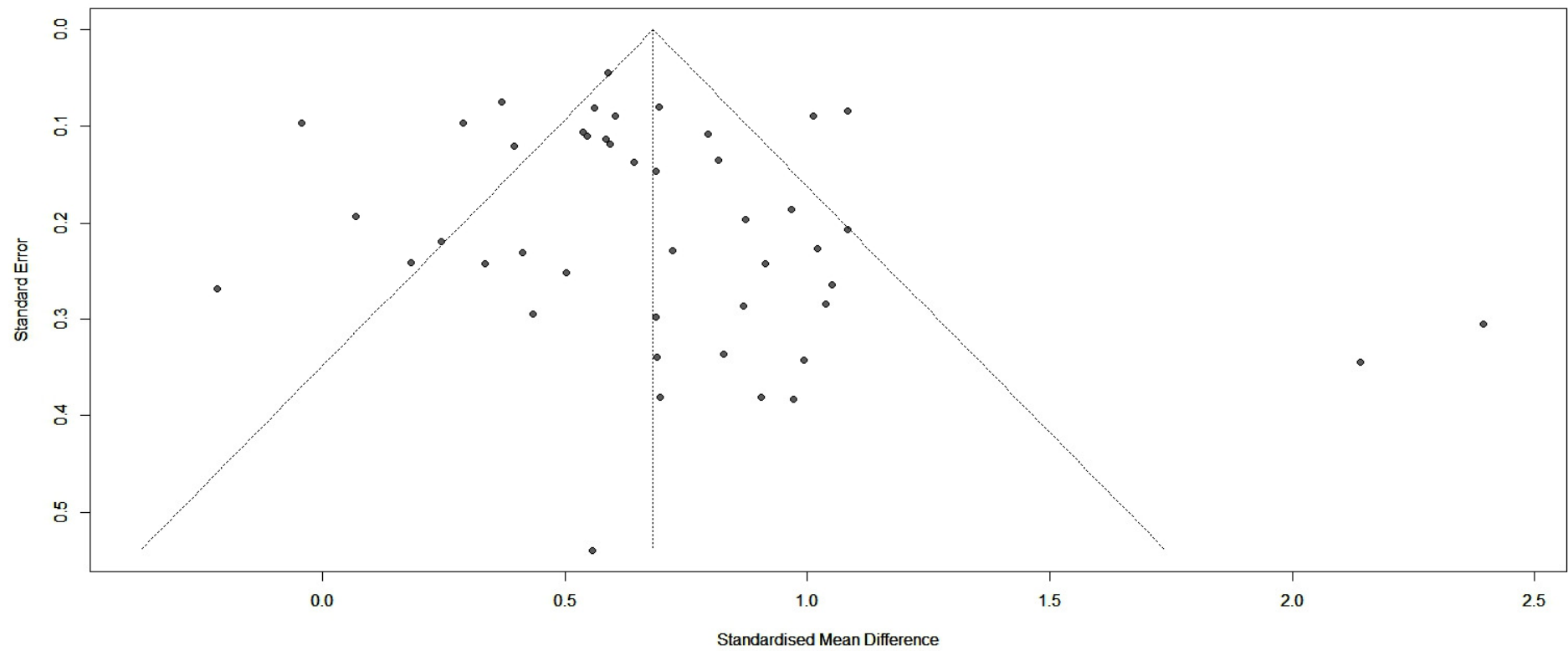

**Supplementary Figure S2:** Funnel plot of GGT values (IU/L)

Supplement: Supplementary file 1 [file jcm-13-03903-s001.zip › Supplementary Figure S2.pdf]
